# A retrospective, longitudinal study of factors associated with new antipsychotic medication use among recently admitted long-term care residents

**DOI:** 10.1186/s12877-015-0127-8

**Published:** 2015-10-19

**Authors:** Andrea Foebel, Anna Ballokova, Nathalie IH Wellens, Daniela Fialova, Koen Milisen, Rosa Liperoti, John P Hirdes

**Affiliations:** Department of Medical Epidemiology and Biostatistics Karolinska Institutet, Nobels väg 12A, Stockholm, Sweden; School of Public Health and Health Systems, University of Waterloo, Waterloo, Canada; Department of Geriatrics and Gerontology, 1st Faculty of Medicine, Charles University, Prague, Czech Republic; Department of Public Health and Primary Care, Center for Health Services and Nursing Research, KULeuven, Leuven, Belgium; Geriatrics Center and Institute of Gerontology, University of Michigan, Ann Arbor, MI USA; Department of Social and Clinical Pharmacy, Faculty of Pharmacy in Hradec Kralove, Charles University, Prague, Czech Republic; Division of Geriatric Medicine, Leuven University Hospital, Herestraat 49, Leuven, 3000 Belgium; Centro Medicina dell’Invecchiamento, Università Cattolica del Sacro Cuore, Rome, Italy

**Keywords:** Long-term care, Mental health, Antipsychotic medications, Risk factors, InterRAI assessment instruments

## Abstract

**Background:**

Use of antipsychotic (AP) medications is high and often inappropriate among institutionalized populations. Little is known about the correlates of new AP drug use following admission to long-term care (LTC) settings. This study investigated the frequency and correlates of new AP drug use among newly admitted LTC residents.

**Methods:**

This longitudinal, retrospective study used data from the interRAI - Nursing Home Minimum Data Set version 2.0 (MDS 2.0) instrument. Data about demographic, clinical and social characteristics, and medication use, were collected in Ontario, Canada, from 2003–2011 by trained nurses. Residents with complete admission and 3–6 month follow-up data were included (*N* = 47,768). Multivariate logistic regression analyses, stratified by gender, explored correlates of new AP drug use upon admission to LTC.

**Results:**

New AP drug users comprised 7 % of the final cohort. Severe cognitive impairment, dementia, and motor agitation were significantly associated with new AP drug use among both sexes. Additionally, behavioural problems, conflicts with staff and reduced social engagement were strong correlates of new AP drug use.

**Conclusions:**

Social factors were as strongly associated with new AP drug use after LTC admission as clinical factors. Strategies to prevent the potential misuse of AP drugs upon LTC admission should consider the social determinants of such prescribing.

## Background

Despite being approved for indications such as schizophrenia and bipolar disorder, antipsychotic (AP) drugs are prescribed off-label for numerous clinical conditions and disorders and are commonly used to treat the behavioural and psychiatric symptoms of dementia (BPSD) [1–3]. Dementia is a progressive, irreversible clinical syndrome, affecting 35.6 million people worldwide [4]. It is characterized by widespread decline in intellectual functions such as memory, communication skills, performing day-to-day activities, reasoning, and changes in social behaviour [5]. More than 80 % of nursing home residents with dementia develop BPSD [6, 7]. BPSD symptoms are characterized by agitation, aggression, restlessness, wandering, shouting, repetitive vocalizations, sleep disturbance, depression and psychosis [8].

AP drugs are commonly prescribed to reduce BPSD despite the lack of evidence about their efficacy, high placebo responses and serious adverse events [9–11]. There is growing international concern about the misuse of psychotropic medications, including AP drugs, as chemical restraints, particularly in institutionalized populations [12–14]. Estimates of AP drug use in nursing home environments range from 20 to 44 % [15–18]. In European nursing homes, this rate was 33 % among residents with dementia [19]. More worryingly, studies have shown that as much as 80 % of this use is among residents without a diagnosis of severe mental illness [15, 16, 18].

Use of atypical AP drugs in patients with dementia can lead to acute and sub-acute side effects, in particular sedation, postural hypotension, and falls, especially at higher doses. Conventional AP drugs can frequently cause serious adverse effects such as extrapyramidal syndrome and tardive dyskinesia [1]. In 2005, both the U.S. Food and Drug Administration (FDA) and Health Canada issued black-box warnings for atypical AP drugs due to increased risk of mortality and cerebrovascular events among patients with dementia [20, 21]. Based on newer evidence, the FDA extended this warning to include conventional AP drugs in 2008 [22]. Similar European recommendations were released by the European Medicines Agency (EMA) in 2008 [23]. The effectiveness of these warnings has been studied. In North America, overall prescription rates of AP drugs to individuals with dementia declined, while overall absolute rates of AP drugs continued to increase [24, 25].

The transition from community to long-term care (LTC) facilities is usually stressful. Only a small proportion of AP drug use upon LTC admission appears to be continuation of prior AP drug use [26]. Thus, it is possible that behavioural changes in response to such transitions could trigger new AP drug prescriptions. Alternately, BPSD could itself be a trigger for institutionalization. Nonetheless, LTC admission and the change in environment could be risk factors for the new (and sometimes excessive) use of AP drugs. The study aimed to estimate the frequency of new AP drug use in residents newly admitted to LTC and to explore socio-demographic and clinical factors associated with such use.

## Methods

### Data source

Data for the study were obtained from provincial repositories at the University of Waterloo, Canada. In Canada, the Canadian Institute for Health Information (CIHI) receives LTC data collected using the interRAI Nursing Home Minimum Data Set version 2.0 (MDS 2.0) instrument through its Continuing Care Reporting System (CCRS) [27]. Ontario, with a population of more than 13,000,000 people, is Canada’s most populous province and the first to mandate interRAI instruments in clinical practice. CIHI ensures that reporting standards are met and performs data quality checks for all interRAI data submissions. After submission, unique identifiers are created to de-identify individuals and allow for linkage with other databases.

This study used data available for research purposes based on existing data-sharing agreements between CIHI, interRAI and the University of Waterloo. interRAI is an international consortium of researchers in more than 30 countries that strives to develop comprehensive, standardized assessment instruments to inform care and improve the quality of life of vulnerable persons in many care settings (www.interrai.org). This research study and the use of anonymized MDS 2.0 data were approved by the University of Waterloo’s Office of Research Ethics.

The MDS 2.0 instrument contains more than 300 items to comprehensively describe resident characteristics, including socio-demographic variables, clinical characteristics, physical and cognitive status, medical diagnoses, major health problems and symptoms, current service use and drug use. MDS diagnostic items have demonstrated good reliability when compared to administrative data for common chronic conditions such as diabetes [28]. The assessment tool contains several standardized functional scales to assess domains such as physical functioning, cognitive status, and overall health status. All of these scales have been validated in nursing home populations [29–32].

When collecting information during assessment, trained assessors, usually nurses, verify information using several sources, including direct observation, interviews with residents, family members and formal service providers, and review of medical records where available. From items embedded within the MDS 2.0, a number of clinical assessment protocols (CAPs) can be generated to assist in care planning. The CCRS data for this study included assessments collected between January 2003 and December 2011 from LTC facilities in Ontario.

### Sample

**Fig. 1:**
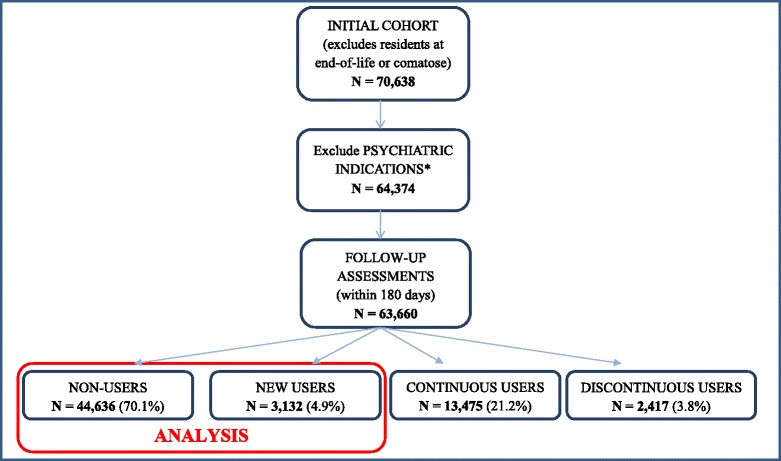
Summary of sample selected from long-term care and complex continuing care residents. *Excludes psychiatric disorders, schizophrenia, Tourette’s syndrome, Huntington’s disease, and hallucinations and individuals receiving antipsychotics for augmentation of anti-depressive therapy

This retrospective longitudinal study explored new use of AP drugs among residents newly admitted to LTC facilities in Ontario between 2003 and 2011. The sample included 70,638 individuals who had complete MDS 2.0 assessment data at baseline and 6-month follow-up assessment. Individuals were excluded if they were comatose, considered to be at the end of life or had no data collected about AP drug use. Residents with neuro-psychiatric conditions including Tourette’s syndrome, Huntington’s disease, schizophrenia or psychiatric disorders, were excluded from the current analysis as these diseases represent labelled indications for antipsychotic treatments. Also, patients receiving antipsychotics for augmentation of antidepressive therapy (FDA approved indication) have been excluded. The detailed sample description is given in Fig. 1.

Baseline and follow up assessments provided comprehensive information about all necessary demographic, clinical and social characteristics of LTC residents, as well as about AP drug use.

### Measuring AP drug use

The MDS 2.0 instrument includes a specific item about the use of AP drugs in the seven days prior to assessment. This information is verified using multiple sources of information including physician order sheets and drug administration records. Based on the data collected in this item at both baseline and follow-up assessments (after a maximum of 180 days), residents were classified into the following groups: non-users (those without AP drug use at baseline and follow-up), new users (those without AP drug use at baseline, but with AP drug use at the follow-up), continuous users (those with AP drug use at both baseline and follow-up), or discontinuous users (those with AP drug use at baseline, but not at follow-up) (see Fig. 1). For the purposes of this study, continuous and discontinuous users were excluded from all analyses. New users of AP drugs and a control group of non-users of AP drugs were considered in regression modelling.

### Other measures

All data on potential correlates of AP drug use were identified from the MDS 2.0 dataset, including calculated functional scales and CAPs. The MDS 2.0 contains an extensive list of disease diagnosis items, allowing assessors to record a number of health conditions as primary or current diagnoses. The presence of dementia (Alzheimer’s and non-Alzheimer’s types), congestive heart failure (CHF), diabetes (both type I and II), and hypertension were determined from this list. Items for other symptoms and conditions, including hearing impairment, visual impairment, presence of urinary tract infections, motor agitation, presence of delusions and pain were also available. Depressive symptoms were identified using both the check-box item for depression and a score of three or more on the Depression Rating Scale (DRS) [32]. During the assessment, information was collected about all medications taken in the previous seven days. The mean number of drugs taken by residents was reported. Specific items describing the use of other psychoactive medications (antidepressants, anxiolytics and hypnotics) allowed these potential correlates to be analyzed.

The Activities of Daily Living (ADL) Hierarchy scale was used to determine the level of functional status impairment [30]. The Changes in Health, End-stage disease and Signs and Symptoms (CHESS) score provided information about instability of the health status [31]. The Cognitive Performance Scale (CPS) incorporates memory impairment, level of consciousness and executive functions into a composite score to assess cognitive status [29]. In each of these scales, higher scores represent increasing levels of impairment or instability [29–32]. The proportions of residents triggering the Communication CAP (signaling communication problems) and Delirium CAP (signaling presence of delirium) were reported and considered as potential correlates of AP drug use.

A number of items in the dataset provided information about social characteristics of LTC residents. The presence of wandering, verbal or physical abuse, resistance to care or socially inappropriate behaviour was used to create a composite item for any behavioural problems. The presence of any conflict with staff or family and friends and reduced social engagement were obtained from relevant items.

For the purposes of this study, any use of trunk, limb or chair restraints from relevant items on the assessment was considered for analyses. Information about whether residents had been hospitalized or whether they visited emergency departments (EDs) in the 90 days prior to assessment was also collected from specific items.

### Statistical analysis

Baseline characteristics of all residents were described according to AP drug use; differences in these characteristics were identified using t-tests for continuous variables and chi-square tests for categorical variables. To examine factors associated with new AP drug use, multivariate logistic regression modelling was performed, with independent variables assessed in LTC residents during the baseline assessment used as potential correlates of new AP drug use. As gender may act as effect modifier in the explored relationships, all analyses were stratified by gender. All baseline characteristics were finally included in regression models which include most of the covariates explored. A decision was made to include ADL and CPS scores instead of CHESS scores in the final models. For the final models, odds ratios (ORs) and associated 95 % confidence intervals (CIs) together with *p*-values were derived. All analyses were conducted using SAS software (version 9.4, SAS Institute Inc., Cary, NC.).

## Results

### Sample

A total of 63,660 newly admitted nursing home residents met the inclusion criteria. Follow-up assessments within 180 days of the admission time were included, but most follow-ups were completed around 90 days in accordance with facility protocols. At admission, 19,024 newly admitted LTC residents (29.9 %) reported AP use and 44,636 (70.1 %) did not. Of individuals not reporting AP use at admission, the frequency of new prescription of AP drugs was 7.0 %. At follow-up, 13,475 residents were continuous users, 3,132 were new users and 2,417 were discontinuous users (see Fig. 1). Only groups of new and non-users of APs (as a control group) were considered for analyses.

### Descriptive characteristics

Non-users of AP drugs were older than new users (proportion of patients younger than 85 years was approximately 47 and 54 %, respectively). Both subgroups were predominantly female (more than 60 %) with two out of three being dependent in ADLs. New users were significantly more cognitively impaired than non-users (CPS > 3 in 62.2 % and 38.7 %, respectively). Behavioural problems were common in general, but more so in the new user group (59.2 %) (see Table 1).

**Table 1:** Demographic and clinical characteristics of newly admitted residents to LTC and CCC facilities in Ontario, Canada according to antipsychotic drug use (*N* = 47,768)

Characteristic		Non-users	New users	*p* value*
Number of residents		*n* = 44,636	*n* = 3,132	
		*n* (%)	*n* (%)	
Demographic				
Age, mean (SD)		83.23 (9.5)	82.51 (8.8)	<0.0001
Under 65		2,152 (4.8)	128 (4.1)	<0.0001
65–74 years		3,685 (8.3)	323 (10.3)	
75–84 years		15,159 (34.0)	1,231 (39.3)	
85+ years		23,640 (53.0)	1,450 (46.3)	
Gender				
Female		30,241 (67.7)	1,902 (60.7)	<0.0001
Clinical				
Functional Scales and CAPS				
ADL Hierarchy Scale^a^	0	4,457 (10.0)	230 (7.3)	<0.0001
	1-2	13,557 (30.4)	1,023 (32.7)	
	3+	26,622 (59.6)	1,879 (60.0)	
CHESS^b^	0	25,085 (56.1)	1,782 (56.9)	0.047
	1-2	17,924 (40.1)	1,211 (38.7)	
	3+	1,654 (3.7)	139 (4.4)	
CPS^c^	0	9,392 (21.0)	242 (7.7)	<0.0001
	1-2	17,960 (40.2)	943 (30.1)	
	3+	17,284 (38.7)	1,947 (62.2)	
Communication CAP		13,819 (31.0)	1,331 (42.5)	<0.0001
Delirium CAP		3,048 (6.8)	338 (10.8)	<0.0001
Comorbidities				
Comorbid Conditions, mean (SD)		4.66 (2.2)	4.34 (2.1)	<0.0001
Any Dementia		21,541 (48.3)	2,337 (74.6)	<0.0001
Congestive Heart Failure		6,538 (14.7)	342 (10.9)	<0.0001
Hypertension		25,770 (57.7)	1,691 (54.0)	<0.0001
Diabetes (type I and/or II)		11,064 (24.8)	658 (21.0)	<0.0001
Other Clinical Symptoms				
Hearing Impairment		16,809 (37.7)	1,152 (36.8)	0.33
Vision Impairment		17,910 (40.1)	1,227 (39.2)	0.30
Pain^d^		20,355 (45.6)	1,116 (35.6)	<0.0001
Motor Agitation		3,172 (7.1)	445 (14.2)	<0.0001
Delusions		418 (0.9)	82 (2.6)	<0.0001
Depressive symptoms^e^		8,111 (18.2)	599 (19.1)	<0.0001
Urinary Tract Infection		3,315 (7.4)	213 (6.8)	0.20
Medication Use				
Number of Medications, mean (SD)		9.44 (4.8)	8.48 (4.7)	<0.0001
Other Psychoactive Medications^f^		20,932 (46.9)	1,611 (51.4)	<0.0001
Social				
Any Behavioural problem^g^		14,416 (32.3)	1,853 (59.2)	<0.0001
Conflict with Staff		1,409 (3.2)	168 (5.4)	<0.0001
Conflict with Family/Friends		1,863 (4.2)	223 (7.1)	<0.0001
Reduced Social Engagement		27,618 (61.9)	1,620 (51.7)	<0.0001
Isolation		1,468 (3.3)	131 (4.2)	0.007
Treatments and Service Use				
Use of restraints		2,916 (6.5)	224 (7.2)	0.18
Hospital Stay		12,626 (28.3)	800 (25.5)	0.001
Emergency Department visits		6,752 (15.1)	461 (14.7)	0.54
Other				
Days between assessments (baseline to follow up) mean (SD)		85.48 (15.6)	85.74 (15.4)	0.002

*CAP*, Clinical Assessment Protocol; *CCC*, Complex Continuing Care; *LTC*, Long-Term Care; *SD*, Standard Deviation

^a^ADL Hierarchy Scale: Activities of Daily Living Scale [range 0-6]: Functionally independent (ADL score of 0); supervision required to limited impairment (ADL score of 1-2); extensive impairment to total dependence (ADL score 3+)

^b^CHESS: Changes in Health, End-stage disease and Signs and Symptoms [range 0-6]: Health stability classified as having no health instability (CHESS score of 0); minimal to low health instability (CHESS score of 1–2); or moderate to very high health instability (CHESS score 3+)

^c^CPS: Cognitive Performance Scale (CPS version 2) [range 0–6]: Intact cognition (CPS score of 0); borderline intact to mild cognitive impairment (CPS score 1–2); moderate to very severe cognitive impairment (CPS score 3+)

^d^Pain Scale: [range 0-4]: Pain classified as any pain (score of 1+)

^e^Depressive symptoms: either presence of depression using the check-box item on the MDS 2.0 or a score of 3 or more on the Depression Rating Scale [range 0-6], which represents probable depressive symptoms

^f^Other Psychoactive Medication includes anti-depressants, hypnotics, or anxiolytics

^g^Any behavioural problems: Composite item for any wandering, verbal or physical abuse, resistance to care or socially inappropriate behaviour

**Table 2:** Correlates of new antipsychotic drug use among newly admitted residents to LTC and CCC facilities in Ontario, Canada (*N* = 47,768) – multivariate logistic regression models

	Model for women (*N* = 32,143)			Model for men (*N* = 15,625)		
	New users *n* = 1,902			New users *n* = 1,230		
	Non-users *n* = 30,241			Non-users *n* = 14,395		
Characteristics	Odds ratio	95 % CI	*p* value	Odds ratio	95 % CI	*p* value
Demographic						
Age (under 75 years)	1.39	(1.19, 1.61)	<0.0001	1.03	(0.88, 1.22)	0.70
Clinical						
ADL^a^ score 1–2	1.03	(0.85, 1.25)	0.33	1.08	(0.84, 1.39)	0.21
ADL score 3+	0.94	(0.78, 1.13)	0.20	0.95	(0.74, 1.22)	0.26
CPS^b^ score 1–2	1.37	(1.13, 1.66)	0.81	1.47	(1.14, 1.89)	0.74
CPS score 3+	1.82	(1.48, 2.25)	<0.0001	2.27	(1.74, 2.97)	<0.0001
Communication CAP	1.05	(0.95, 1.17)	0.34	1.04	(0.91, 1.19)	0.57
Delirium CAP	1.26	(1.07, 1.47)	0.004	1.20	(0.99, 1.47)	0.07
Any Dementia	1.89	(1.67, 2.13)	<0.0001	2.23	(1.91, 2.59)	<0.0001
Congestive Heart Failure	0.90	(0.77, 1.05)	0.19	0.97	(0.80, 1.17)	0.73
Hypertension	1.03	(0.93, 1.14)	0.55	0.92	(0.81, 1.04)	0.20
Diabetes (type I and/or II)	0.90	(0.80, 1.02)	0.10	0.93	(0.80, 1.07)	0.32
Hearing Impairment	1.01	(0.91, 1.11)	0.93	0.83	(0.73, 0.94)	0.004
Vision Impairment	0.86	(0.78, 0.95)	0.003	0.95	(0.84, 1.08)	0.46
Pain^c^	0.89	(0.84, 0.95)	0.0007	0.91	(0.83, 0.99)	0.03
Motor Agitation	1.24	(1.08, 1.44)	0.003	1.45	(1.21, 1.74)	<0.0001
Delusions	1.60	(1.18, 2.17)	0.002	1.88	(1.21, 2.93)	0.005
Depressive symptoms^d^	0.73	(0.64, 0.82)	<0.0001	0.57	(0.47, 0.69)	<0.0001
Urinary Tract Infection	0.99	(0.83, 1.18)	0.88	0.98	(0.89, 1.08)	0.68
Number of Medications	0.94	(0.88, 1.01)	0.11	0.95	(0.72, 1.25)	0.70
Other Psychoactive Medications ^e^	1.68	(1.50, 1.88)	<0.0001	1.66	(1.44, 1.92)	<0.0001
Social						
Any Behavioural Problems^f^	1.91	(1.71, 2.12)	<0.0001	2.15	(1.88, 2.47)	<0.0001
Conflict with Staff	1.61	(1.29, 2.01)	<0.0001	0.98	(0.72, 1.34)	0.90
Conflict with Family/Friends	1.34	(1.10, 1.63)	0.003	1.44	(1.10, 1.87)	0.008
Reduced Social Engagement	0.78	(0.71, 0.87)	<0.0001	0.94	(0.83, 1.06)	0.32
Isolation	0.88	(0.68, 1.15)	0.35	1.11	(0.85, 1.46)	0.45
Treatments and Service Use						
Use of Restraints	0.84	(0.69, 1.02)	0.07	0.85	(0.67, 1.07)	0.17
Hospital Stay	1.05	(0.93, 1.19)	0.44	0.87	(0.74, 1.02)	0.09
Emergency Department Visits	0.98	(0.84, 1.15)	0.79	1.24	(1.02, 1.50)	0.03
Other						
Days between Assessments	1.00	(0.99, 1.00)	0.80	1.00	(1.00, 1.00)	0.70

*CAP*, Clinical Assessment Protocol; *CI*, confidence interval

^a^ADL: Activities of Daily Living hierarchy scale score – reference = 0

^b^CPS: Cognitive Performance Scale – reference = 0

^c^Reference = no pain on the Pain Scale

^d^Score of 3 or higher on the Depression Rating Scale versus score of 0

^e^Includes anti-depressants, hypnotics, and anxiolytics

^f^Presence of any wandering, verbal or physical abuse, resistance to care or socially inappropriate behaviour

Compared to non-users, residents with new AP drug use reported more communication problems and had higher rates of dementia, depressive symptoms, delusions, and motor agitation (see Table 1). However, prevalence of other comorbidities such as CHF, diabetes, and hypertension were lower among new users compared to non-users. Behavioural problems among new AP drug users, and more specifically rates of conflict with staff and family/friends, were higher than those of non-users. Reduced social engagement, on the contrary, was lower among the new user group.

There was no difference in restraint use between new and non-users, but new users reported more isolation. Health status instability (as measured by the CHESS scores) was somewhat higher in the new user group, as was presence of delirium. No differences were observed in prevalence of vision or hearing impairments and the mean number of medications used in both groups was high. The use of other psychoactive medications (antidepressants, anxiolytics, or hypnotics) was high overall, but higher in the new user group compared to non-users. Hospital stays were more common in the non-user group, but ED visits and urinary tract infections were comparable between groups.

### Correlates of new AP drug use

Two separate models were developed to explore correlates of new AP drug use; one for women and one for men (see Table 2). The model for women shows that younger residents were more likely to receive AP drugs within 180 days of admission (OR 1.39; 95 % CI 1.19 – 1.61; *p* < 0.0001). Severe cognitive impairment (OR 1.82; 95 % CI 1.48– 2.25; *p* < 0.0001), and dementia (OR 1.89; 95 % CI 1.67-2.13; *p* < 0.0001) were the strongest clinical correlates of new AP drug use. Presence of delusions (OR 1.60; 95 % CI 1.18- 2.17; *p* = 0.002), delirium (OR 1.26; 95 % CI 1.07– 1.47; *p* = 0.004), and motor agitation (OR 1.24; 95 % CI 1.08-1.44; *p* = 0.003) all increased the likelihood of new use of AP drugs. Concomitant use of other psychoactive medications was also associated with higher likelihood of new AP drug use (OR 1.68; 95 % CI 1.50–1.88; *p* < 0.0001). Depressive symptoms were associated with lower likelihood of new AP use (OR 0.73; 95 % CI 0.64–0.82; <0.0001). Social factors were also found to be strong correlates of new AP drug use. Compared to non-users with similar characteristics, women who became new users were nearly twice as likely to exhibit any behaviour problem (OR 1.91; 95 % CI 1.71–2.12; *p* < 0.0001). Also, conflicts with staff and family/friends (OR 1.61; 95 % CI 1.29–2.01; p < 0.0001 and OR 1.34; 95 % CI 1.10–1.63; p = 0.003; respectively) increased the likelihood of new AP drug use, while reduced social engagement was associated with lower risk (OR 0.78; 95 % CI 0.71–0.87; p < 0.0001). Having pain or visual impairment reduced the likelihood of new AP drug use (OR 0.89; 95 % CI 0.84–0.95; p = 0.0007 and OR 0.86; 95 % CI 0.78–0.95; p = 0.003, respectively).

In general, the model for men is similar to the one for women. Unlike among women, age and delirium were not significant correlates of new AP use. Cognitive impairment (OR 2.27; 95 % CI 1.74–2.97; *p* < 0.0001), dementia (OR 2.23; 95 % CI 1.91–2.59; *p* < 0.0001) and any behavioural problem (OR 2.15; 95 % CI 1.88–2.47; *p* < 0.0001) were all strong correlates of new AP drug use, and these effects were stronger among men than women. The same trend was shown for delusions (OR 1.88; 95 % CI 1.21–2.93; p = 0.005) and motor agitation (OR 1.45; 95 % CI 1.21–1.74; p < 0.0001). Similar to women, use of other psychoactive medications was associated with higher likelihood of new AP drug use (OR 1.66; 95 % CI 1.44–1.92; *p* < 0.0001), while depressive symptoms were associated with lower likelihood (OR 0.57; 95 % CI 0.47, 0.69). Conflict with family and friends (OR 1.44; 95 % CI 1.10–1.87; p = 0.008), but not with staff, was also positively associated with new AP drug use. Hearing (OR 0.83; 95 % CI 0.73–0.94; p = 0.004), but not vision impairment, was associated with reduced likelihood of new AP drug use in men as was the occurrence of pain (OR 0.91; 95 % CI 0.83–0.99; p = 0.03). ED visits were also significant correlates of new AP drug use (OR 1.24; 95 % CI 1.02–1.50; *p* = 0.03), but previous hospital stays were not.

## Discussion

In this large cohort of newly admitted nursing home residents, 7 % became new AP drug users in the six months following admission. This work has built upon previous findings by identifying characteristics associated with new AP drug use, with some interesting results. Common correlates were identified for both men and women, for example, the presence of dementia and cognitive impairment being significantly associated with new use of AP drugs. However, other triggers were also identified. The comprehensiveness of the MDS 2.0 data allowed for the possibility to analyze a substantial number of novel potential correlates, setting this study apart from earlier work. Importantly, this work also found that behavioural and social factors, such as behavioural problems and conflicts with both staff and family and friends, were as strongly correlated with new AP drug use as clinical factors.

The overall rate of AP drug use in this study was 26.1 % for new and continuous users combined. Since this rate excludes individuals with psychiatric conditions, it likely underestimates the actual rates of AP use in these facilities. Nonetheless, it is comparable to other Canadian and American studies [15–17]. In one Canadian study which aimed to identify the variability of prevalence of AP drug use in LTC settings, the overall rate of AP drug prescribing was 32.4 % [17]. Another American study in LTC facilities estimated a mean of 30 % residents receiving at least one AP medication [15]. A study by Gellad and colleagues showed an overall rate of 25.7 % of AP drug use among long-stay nursing home residents [16]. These rates of AP drug use are all higher than those reported in studies from LTC settings in the United Kingdom and Northern Ireland, which reported prevalence rates of AP drug use of 21 % and 20.3 %, respectively [18, 26]. Thus, it would seem that the current findings are in agreement with current Canadian studies even though the data used were not census-level.

Recent work by Maguire and colleagues has explored new use of AP drugs in the period of transition from community to institutional settings, showing that AP drug use increased during this transition time, likely reflecting institutional prescribing cultures [26]. However, the rate of new AP use reported in this study was 16.9 % within the six month period after admission [26]. While the current study found a much lower rate (7 %), it may be because AP drug use is more pervasive in both community and institutional settings in the North American context or alternately, that shorter-term AP use was not captured between assessments.

While it was not the purpose of this paper to explore the appropriateness of new AP prescribing in this sample, the exclusion criteria were chosen to exclude individuals with a clear indication for AP drug use. Also, dementia was found to be associated with an increased likelihood of new AP drug use, but both the FDA and EMA have issued official warnings to limit such use [33, 34]. Interestingly, individuals receiving other psychoactive medications were more likely to begin to use AP drugs, signaling a clustered prescribing pattern. Thus, these findings may give also some insight into the actual patterns of prescribing in institutional settings. This work has many important implications for both practice and public health policy. Findings of this study suggest the presence of temporary indications of AP drug prescription among newly admitted residents, such as cognitive impairment, BPSD and delirium. In particular, as the use of AP drugs is studied at an important point of transition in care (admission to LTC), there may be a greater potential for interventions based on these findings.

To date, no work has focused on correlates of new use of AP drugs at the time of admission. The current findings, that behavioural and social factors, as well as clinical factors significantly influence new prescription of AP drugs after LTC admission, are novel. These correlates can act as signals to identify individuals at risk of potentially inappropriate AP drug prescription upon LTC admission, providing a method for targeting interventions to reduce the potential misuse of AP drugs. Previous work has found that higher AP drug use is linked to staff distress and lower staff-to-patient ratios [35], but these factors could not be explored in the current study.

There are a number of important implications from this work. Firstly, these medications appear to be prescribed more frequently in individuals with dementia, likely to control associated BPSD. Such prescribing should be closely examined, as warnings against the misuse of AP drugs in this indication have been issued by both the FDA and EMA [33, 34]. Further, different national guidelines including those issued by National Institute for Health and Care Excellence stated that these medications should be used maximally for 6 to 12 weeks to treat behavioural symptoms [36]. This study could not explore whether medications were discontinued after 6 or 12 month periods. Nonetheless, it will be important follow up work to determine methods to reduce prescribing and promote appropriate review of AP drugs as recommended.

From a geriatric care perspective, these findings indicate that AP drugs may be used to manage behaviours or cognitive changes such as delusions. Some researchers have suggested that institutional settings should provide better mental health care [26] and these findings support this viewpoint. Other modalities for treating mental health issues in this cohort, such as psychosocial and psychotherapeutic interventions should be explored [1, 8]. Finally, this study focuses on a key transition period – the entry to institutional care. This transition time represents a key window of opportunity for medication reviews, new assessments, monitoring and follow-up. It is possible that medication review at this time led to appropriate new AP use, as people entering institutional environments may be particularly amenable to improvements in their care. However, it is also possible that this new use was not appropriate. This work has helped to identify a population potentially at risk for inappropriate care (namely AP drug use) and a prime target group for interventions.

A number of limitations to this study should be mentioned. Firstly, the sample was a convenience sample from facilities in Ontario that use the MDS 2.0. Thus, data are not census-level and there is the chance of selection bias towards facilities that are most motivated to comprehensively assess and manage residents. However, rates of AP drug use reported here align with earlier Canadian estimates [15, 17]. Next, it is possible that individuals may be prescribed AP drugs immediately upon entry to the facility, meaning they could be counted as continuous rather than new users. This would underestimate the new AP prescription rate and possible reduce the magnitude of the associations observed. Further, medication data were limited to those available from the Canadian MDS 2.0 instrument. Only AP drug use and use of hypnotics, antidepressants and anxiolytics in the past seven days are recorded and information about adherence is not captured. However, given that this study was done in institutional environments, it may be reasonable to expect high adherence rates. These data limitations meant that it was not possible to explore medication use between assessments or to verify that new AP drug use is sustained, and thus the duration of treatment was not explored. The dataset did not allow in-depth exploration of AP drug use, specific ingredients or whether such use was on regular or as-needed (PRN) basis. Thus it was not possible to distinguish between different types of AP drugs and their association with prescription risk factors. There was no information available about facility characteristics such as the number of beds or staffing rates and these correlates could not be explored. This would be worthwhile future work. Finally, it was not the aim of this study to explore potential changes in practice towards AP use following changes in policy, for example, the FDA warning in 2008. The sample size in the current study would not permit such comparisons to be made, but this would be another area that would be important for future work.

Some of the strengths of this study included the large sample size and the comprehensiveness of the data source. Even with lower rates of new AP drugs prescription, the sample size was large enough to allow exploration of correlates of AP drug use. Further, with the MDS 2.0 data, this study explored a number of important potential correlates including behavioural and social factors that are not often available from administrative data sets. Finally, by using a narrow window of transition, risk factors for new AP drug use at an important transition time were identified, making targeted interventions possible.

## Conclusions

Overall, this work has shown that in addition to clinical factors, behaviours and social characteristics were strongly associated with new AP drug use upon admission to institutional care. Such knowledge can provide a first insight into prescribing practices within nursing home environments and identify targets for interventions to increase the quality of care afforded to older people. Reducing inappropriate use of APs and other psychotropic medications is a goal for all facilities and this work has characterized a particularly vulnerable sub-population of new residents at a higher risk of new AP prescription. Interventions to improve care for these subpopulations and improve the overall mental health care in institutional settings can utilize such information to further increase the quality of care.

## Abbreviations

ADL: Activities of daily living
AP: Antipsychotic
BPSD: Behavioural and psychiatric symptoms of dementia
CAP: Clinical assessment protocol
CCRS: Continuing care reporting system
CHESS: Changes in health, end-stage disease and signs and symptoms
CHF: Congestive heart failure
CI: Confidence intervals
CIHI: Canadian institute for health information
CPS: Cognitive performance scale
DRS: Depression rating scale
ED: Emergency department
EMA: European medicines agency
FDA: U.S. food and drug agency
LTC: Long-term care
MDS 2.0: interRAI nursing home minimum data set version 2.0
OR: Odds ratio
PRN: Pro re nata (as needed)

## References

[1] Jeste VD, Blazer D, Casey D, Meeks T, Salzman C, Schneider L (2008). ACNP white paper: update on use of antipsychotic drugs in elderly persons with dementia. Neuropsychopharmacology.

[2] McGrath AM, Jackson GA (1996). Survey of prescribing in residents of nursing homes in Glasgow. BMJ.

[3] Margallo-Lana M, Swann A, O’Brien J, Fairbairn A, Reichelt K, Potkins D (2001). Prevalence and pharmacological management of behavioural and psychological symptoms amongst dementia sufferers living in care environments. Int J Geriatr Psychiatr.

[4] Steinberg M, Shao H, Zandi P, Lyketsos CG, Welsh-Bohmer KA, Norton MC (2008). Point and 5-year period prevalence of neuropsychiatric symptoms in dementia: the Cache County Study. Int J Geriatr Psychiatry.

[5] Scottish Intercollegiate Guideline Network (2006). Management of patients with dementia: a national clinical guideline.

[6] Testad I, Aasland AM, Aarsland D (2007). Prevalence and correlates of disruptive behavior in patients in Norwegian nursing homes. Int J Geriatr Psychiatry.

[7] Zuidema SU, Derksen E, Verhey FR, Koopmans RT (2007). Prevalence of neuropsychiatric symptoms in a large sample of Dutch nursing home patients with dementia. Int J Geriatr Psychiatry.

[8] Richter T, Meyer G, Mohler R, Kopke S (2012). Psychosocial interventions for reducing antipsychotic medication in care home residents. Cochrane Database Syst Rev.

[9] DeDeyn PP, Rabheru K, Rasmussen A, Bocksberger JP, Dautzenberg PL, Eriksson S (1999). A randomised trial of risperidone, placebo and haloperidol for behavioural symptoms of dementia. Neurol.

[10] Street JS, Clark WS, Gannon KS, Cummings JL, Bymaster FP, Tamura RN (2000). Olanzapine treatment of psychotic and behavioural symptoms in patients with Alzheimer’s disease in nursing care facilities: a double-blind randomised, placebo-controlled trial. Arch Gen Psychiatr.

[11] Sink KM, Holden KF, Yaffe K (2005). Pharmacological treatment of neuropsychiatric symptoms of dementia: a review of the evidence. JAMA.

[12] Mann E, Köpke S, Haastert B, Pitkälä K, Meyer G (2009). Psychotropic medication use among nursing home residents in Austria: A cross-sectional study. BMC Geriatr.

[13] Oborne AC, Hooper R, Li KC, Swift CG, Jackson SH (2002). An indicator of appropriate neuroleptic prescribing in nursing homes. Age Ageing.

[14] Feng Z, Hirdes JP, Smith TF, Finne-Soveri H, Chi I, Du Pasuqier JN (2009). Use of physical restraints and antipsychotic medications in nursing homes: A cross-national study. Int J Geriatr Psychiatry.

[15] Chen Y, Briesacher BA, Field TS, Tjia J, Lau DT, Gurwitz JH (2010). Unexplained variation across US nursing homes in antipsychotic prescribing rates. Arch Intern Med.

[16] Gellad WF, Aspinall SL, Handler SM, Stone RA, Castle N, Semla TP (2012). Use of antipsychotics among older residents in Veterans Administration nursing homes. Med Care.

[17] Rochon PA, Stukel TA, Bronskill SE, Gomes T, Sykora K, Wodchis WP (2007). Variation in nursing home antipsychotic prescribing rates. Arch Intern Med.

[18] Shah S, Carey I, Harris T, Dewilde S, Cooke TG. Antipsychotic prescribing to older people living in care homes and the community in England and Wales. Int J Geriatr Psychiatry. 2011;26(4):423–3. 10.1002/gps.2557 20878663

[19] Foebel AD, Liperoti R, Onder G, Finne-Soveri H, Henrard JC, Lukas A, et al. Use of antipsychotic drugs among residents with dementia in European long-term care facilities: Results from the SHELTER Study. J Am Med Dir Assoc. 2014;15(12):911–7 [Epub ahead of print]. 10.1016/j.jamda.2014.07.012 25262195

[20] Food and Drug Agency. Public Health Advisory: Deaths with antipsychotics in elderly patients with behavioural disturbances. FDA. 2005. http://www.fda.gov/Safety/MedWatch/SafetyInformation/SafetyAlertsforHumanMedicalProducts/ucm150688.htm. Accessed 22 Jun 2014.

[21] Health Canada. Health Canada Advises Consumers about Important Safety Information on Atypical Antipsychotic Drugs and Dementia. Health Canada. 2005. http://www.healthycanadians.gc.ca/recall-alert-rappel-avis/hc-sc/2005/13696a-eng.php. Accessed 24 Jun 2014.

[22] Food and Drug Agency. Antipsychotics, Conventional and Atypical. FDA. 2008. http://www.fda.gov/Safety/MedWatch/SafetyInformation/SafetyAlertsforHumanMedicalProducts/ucm110212.htm. Accessed 22 Jun 2014.

[23] European Medicines Agency. CHMP Assessment Report on Conventional Antipsychotics. EMA. 2008. http://www.ema.europa.eu/docs/en_GB/document_library/Report/2010/01/WC500054057.pdf. Accessed 24 Jun 2014.

[24] Valiyeva E, Herrmann N, Rochon PA, Gill SS, Anderson GM (2008). Effect of regulatory warning on antipsychotic prescription rates among elderly patients with dementia: a population-based time-series analysis. CMAJ.

[25] Dorsey ER, Rabbani A, Gallagher SA, Conti RM, Alexander GC (2010). Impact of FDA black box advisory on antipsychotic medication use. Arch Intern Med.

[26] Maguire A, Hughes C, Cardwell C, O’Reilly D (2013). Psychotropic medications and the transition into care: A national data linkage study. J Am Geriatr Soc.

[27] Canadian Institute for Health Information. Information about Continuing Care. CIHI. http://www.cihi.ca/CIHI-ext-portal/internet/EN/TabbedContent/types+of+care/hospital+care/continuing+care/cihi018109. Accessed 24 Jun 2014.

[28] Foebel AD, Hirdes JP, Heckman GA, Kergoat MJ, Patten S, Marrie RA, et al. Diagnostic data for neurological conditions in interRAI assessments in home care, nursing home and mental health care settings: a validity study. BMC Health Serv Res. 2013;13:457. 10.1186/1472-6963-13-457 PMC3893477 24176093

[29] Morris JN, Fries BE, Mehr DR, Hawes C, Phillips C, Mor V (1994). MDS Cognitive Performance Scale. J Gerontol..

[30] Morris JN, Fries BE, Morris SA (1999). Scaling ADLs within the MDS. J Gerontol A Biol Sci Med Sci.

[31] Hirdes JP, Frijters DH, Teare GF (2003). The MDS-CHESS scale: a new measure to predict mortality in institutionalized older people. J Am Geriatr Soc.

[32] Burrows A, Morris JN, Simon S, Hirdes JP, Phillips C (2000). Development of a Minimum Data Set-Based Depression Rating Scale for use in nursing homes. Age Ageing.

[33] Food and Drug Agency. Public Health Advisory: Deaths with antipsychotics in elderly patients with behavioural disturbances, 2011 update. FDA. 2011. http://www.fda.gov/drugs/drugsafety/postmarketdrugsafetyinformationforpatientsandproviders/ucm124830.htm. Accessed 9 Jun 2014.

[34] European Medicines Agency. EMEA 2010 Priorities for Drug Safety Research: Safety aspects of antipsychotics in demented patients. EMA. 2009. http://www.ema.europa.eu/docs/en_GB/document_library/Other/2010/03/WC500076323.pdf. Accessed 9 Jun 2014.

[35] Zuidema SU, De Jonghe JFM, Verhey FRJ, Koopmans RT (2011). Psychotropic drug prescription in nursing home patients with dementia: Influence of environmental correlates and staff distress on physicians’ prescription behaviour. Int Psychogeriatr.

[36] National Institute for Health and Care Excellence. Dementia: Supporting people with dementia and their carers in health and social care. NICE. 2006. http://www.nice.org.uk/guidance/cg42/resources/guidance-dementia-pdf. Accessed 10 Jul 2014.

